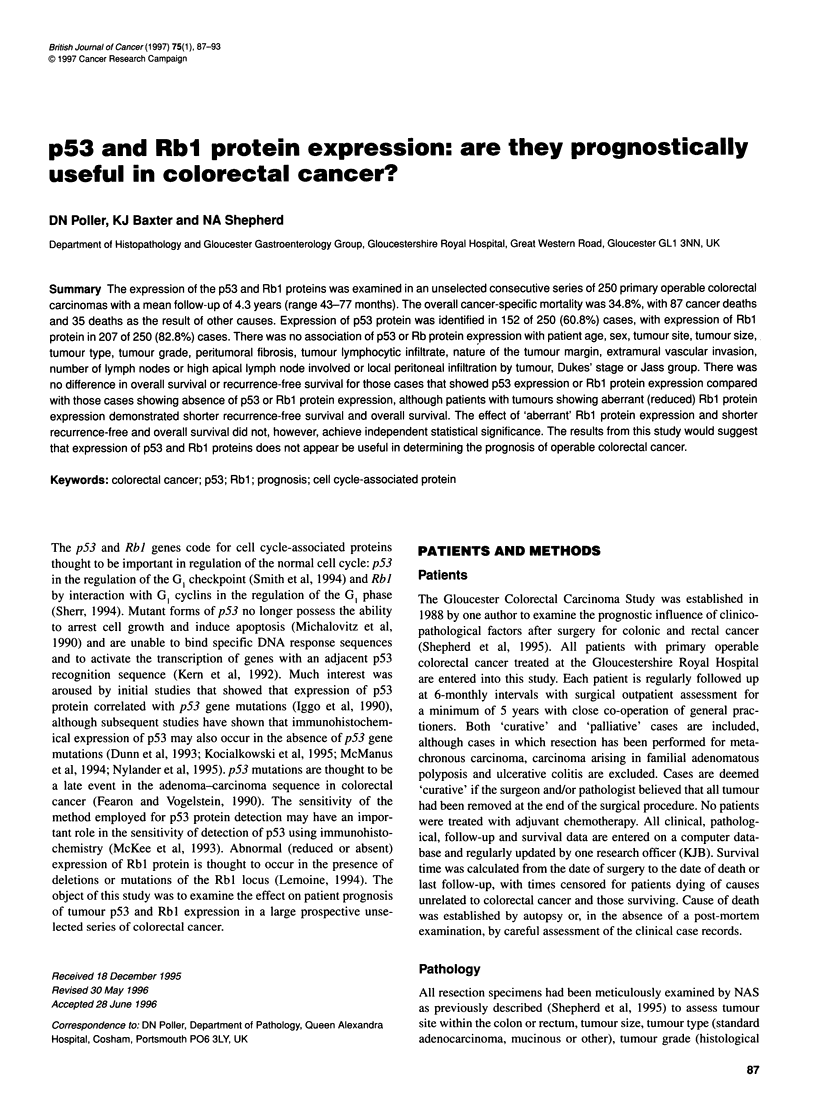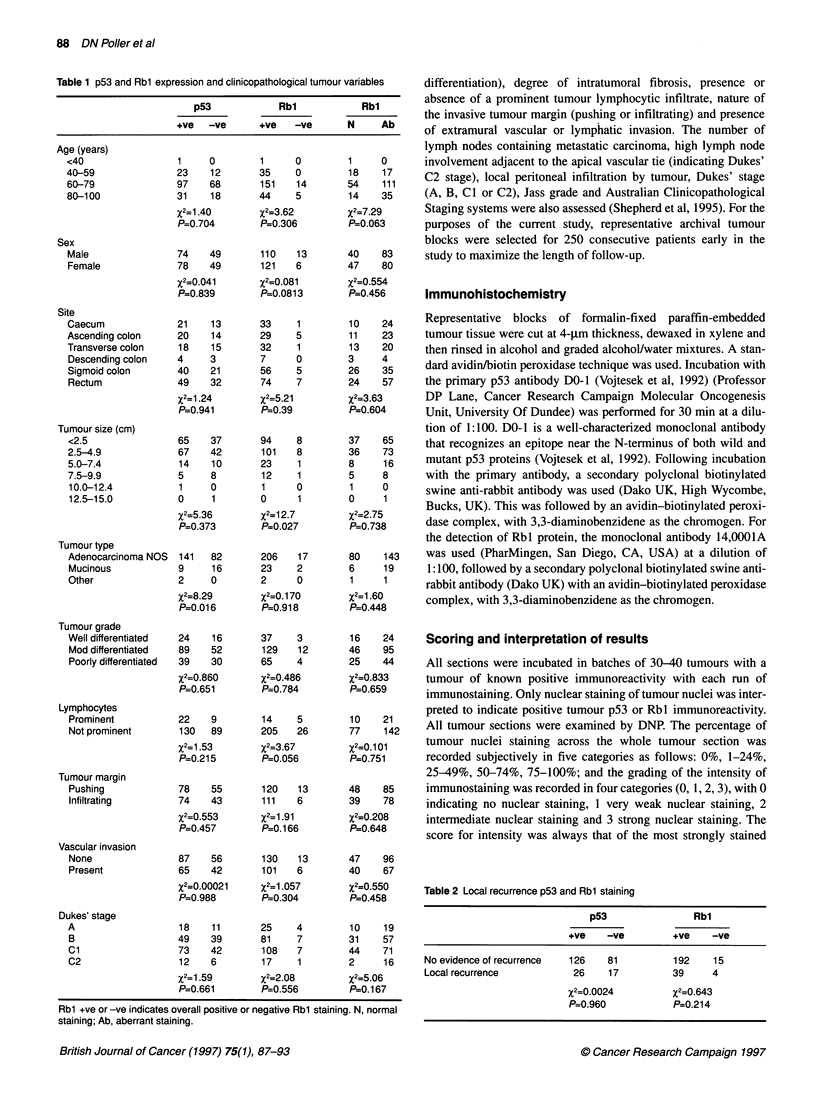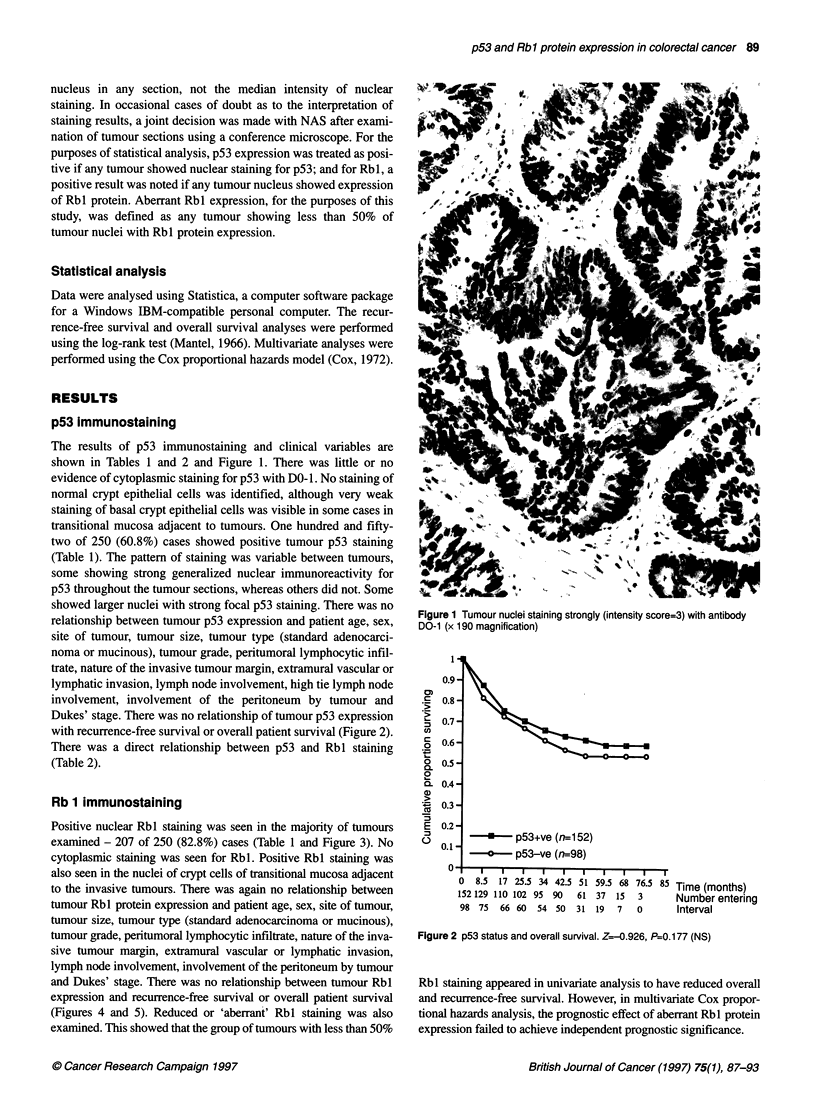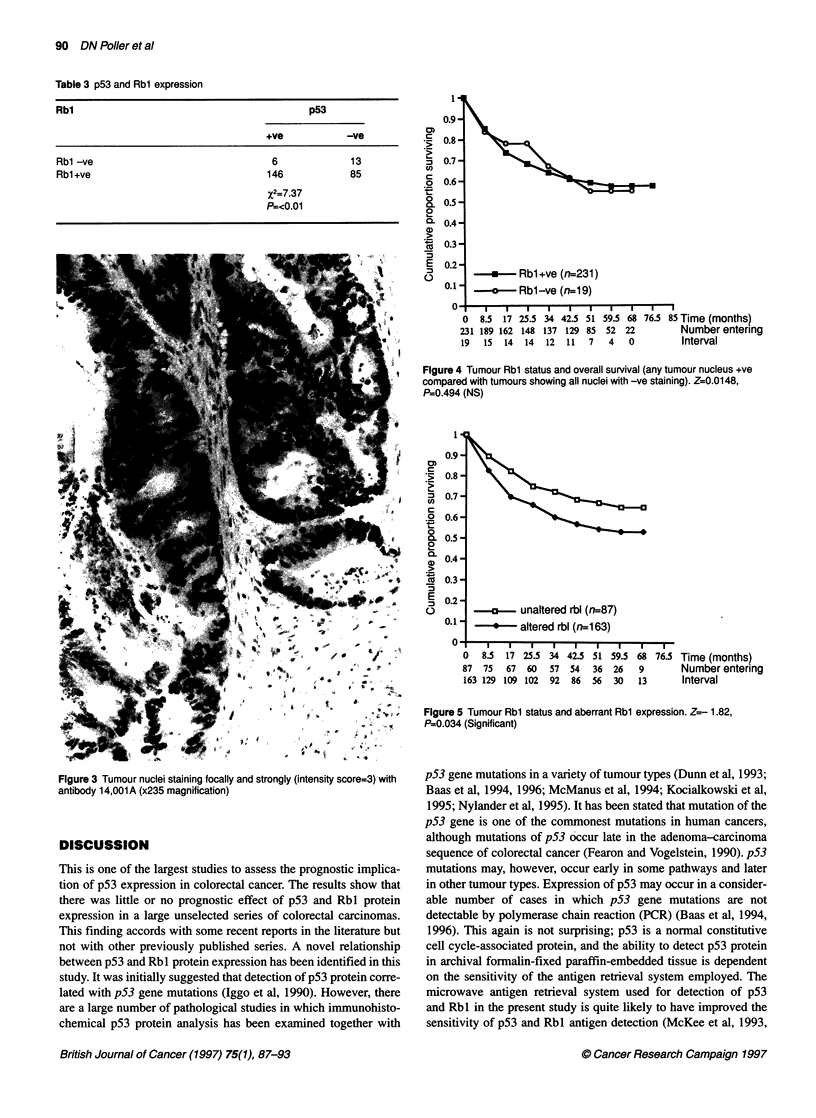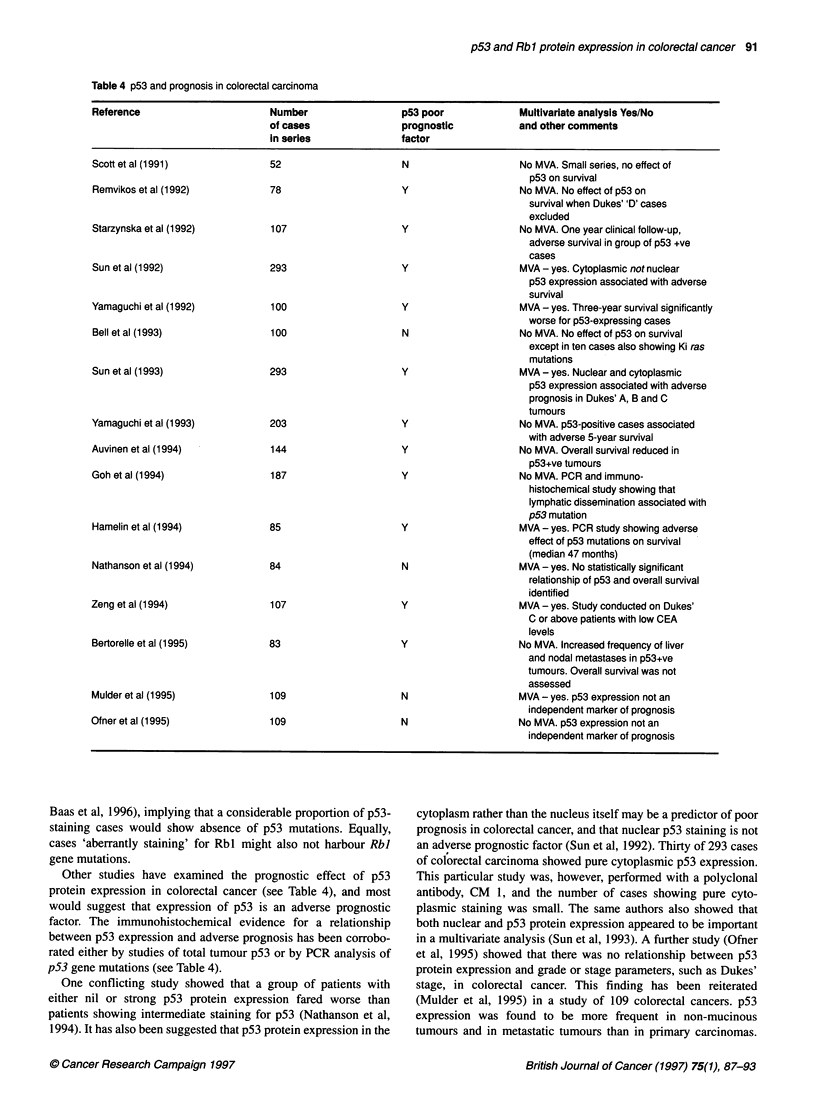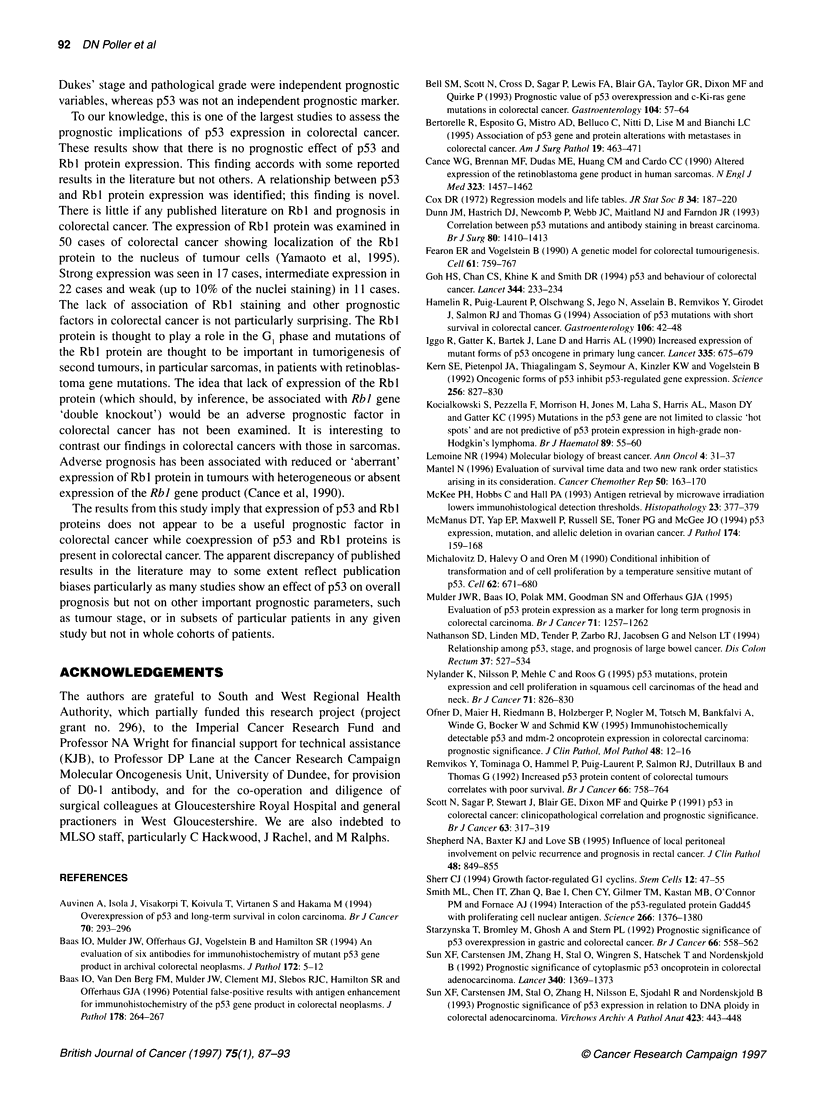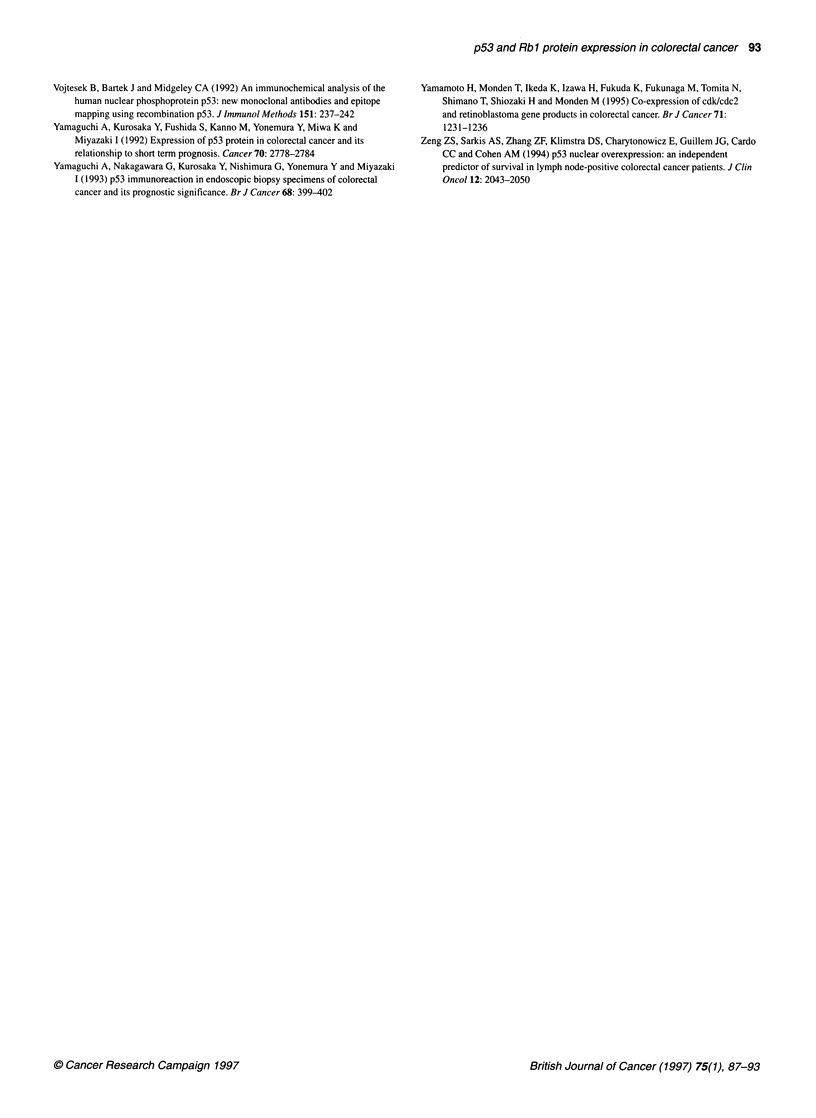# p53 and Rb1 protein expression: are they prognostically useful in colorectal cancer?

**DOI:** 10.1038/bjc.1997.14

**Published:** 1997

**Authors:** D. N. Poller, K. J. Baxter, N. A. Shepherd

**Affiliations:** Department of Histopathology and Gloucester Gastroenterology Group, Gloucestershire Royal Hospital, UK.

## Abstract

**Images:**


British Journal of Cancer (1997) 75(1), 87–93
© 1997 Cancer Research Campaign

### p53 and Rb1 protein expression: are they prognostically useful in colorectal cancer?

**DN Poller, KJ Baxter and NA Shepherd**

Department of Histopathology and Gloucester Gastroenterology Group, Gloucestershire Royal Hospital, Great Western Road, Gloucester GL1 3NN, UK

**Summary** The expression of the p53 and Rb1 proteins was examined in an unselected consecutive series of 250 primary operable colorectal carcinomas with a mean follow-up of 4.3 years (range 43–77 months). The overall cancer-specific mortality was 34.8%, with 87 cancer deaths and 35 deaths as the result of other causes. Expression of p53 protein was identified in 152 of 250 (60.8%) cases, with expression of Rb1 protein in 207 of 250 (82.8%) cases. There was no association of p53 or Rb protein expression with patient age, sex, tumour site, tumour size, tumour type, tumour grade, peritumoral fibrosis, tumour lymphocytic infiltrate, nature of the tumour margin, extramural vascular invasion, number of lymph nodes or high apical lymph node involved or local peritoneal infiltration by tumour, Dukes' stage or Jass group. There was no difference in overall survival or recurrence-free survival for those cases that showed p53 expression or Rb1 protein expression compared with those cases showing absence of p53 or Rb1 protein expression, although patients with tumours showing aberrant (reduced) Rb1 protein expression demonstrated shorter recurrence-free survival and overall survival. The effect of 'aberrant' Rb1 protein expression and shorter recurrence-free and overall survival did not, however, achieve independent statistical significance. The results from this study would suggest that expression of p53 and Rb1 proteins does not appear be useful in determining the prognosis of operable colorectal cancer.

**Keywords:** colorectal cancer; p53; Rb1; prognosis; cell cycle-associated protein

The *p53* and *Rb1* genes code for cell cycle-associated proteins thought to be important in regulation of the normal cell cycle: *p53* in the regulation of the $G_1$ checkpoint (Smith et al, 1994) and *Rb1* by interaction with $G_1$ cyclins in the regulation of the $G_1$ phase (Sherr, 1994). Mutant forms of *p53* no longer possess the ability to arrest cell growth and induce apoptosis (Michalovitz et al, 1990) and are unable to bind specific DNA response sequences and to activate the transcription of genes with an adjacent p53 recognition sequence (Kern et al, 1992). Much interest was aroused by initial studies that showed that expression of p53 protein correlated with *p53* gene mutations (Iggo et al, 1990), although subsequent studies have shown that immunohistochemical expression of p53 may also occur in the absence of *p53* gene mutations (Dunn et al, 1993; Kocialkowski et al, 1995; McManus et al, 1994; Nylander et al, 1995). *p53* mutations are thought to be a late event in the adenoma–carcinoma sequence in colorectal cancer (Fearon and Vogelstein, 1990). The sensitivity of the method employed for p53 protein detection may have an important role in the sensitivity of detection of p53 using immunohistochemistry (McKee et al, 1993). Abnormal (reduced or absent) expression of Rb1 protein is thought to occur in the presence of deletions or mutations of the Rb1 locus (Lemoine, 1994). The object of this study was to examine the effect on patient prognosis of tumour p53 and Rb1 expression in a large prospective unselected series of colorectal cancer.

*Received 18 December 1995*
*Revised 30 May 1996*
*Accepted 28 June 1996*

*Correspondence to:* DN Poller, Department of Pathology, Queen Alexandra Hospital, Cosham, Portsmouth PO6 3LY, UK

### PATIENTS AND METHODS

### Patients

The Gloucester Colorectal Carcinoma Study was established in 1988 by one author to examine the prognostic influence of clinicopathological factors after surgery for colonic and rectal cancer (Shepherd et al, 1995). All patients with primary operable colorectal cancer treated at the Gloucestershire Royal Hospital are entered into this study. Each patient is regularly followed up at 6-monthly intervals with surgical outpatient assessment for a minimum of 5 years with close co-operation of general practitioners. Both 'curative' and 'palliative' cases are included, although cases in which resection has been performed for metachronous carcinoma, carcinoma arising in familial adenomatous polyposis and ulcerative colitis are excluded. Cases are deemed 'curative' if the surgeon and/or pathologist believed that all tumour had been removed at the end of the surgical procedure. No patients were treated with adjuvant chemotherapy. All clinical, pathological, follow-up and survival data are entered on a computer database and regularly updated by one research officer (KJB). Survival time was calculated from the date of surgery to the date of death or last follow-up, with times censored for patients dying of causes unrelated to colorectal cancer and those surviving. Cause of death was established by autopsy or, in the absence of a post-mortem examination, by careful assessment of the clinical case records.

### Pathology

All resection specimens had been meticulously examined by NAS as previously described (Shepherd et al, 1995) to assess tumour site within the colon or rectum, tumour size, tumour type (standard adenocarcinoma, mucinous or other), tumour grade (histological

**Table 1** p53 and Rb1 expression and clinicopathological tumour variables

| | p53 | | Rb1 | | Rb1 | |
|---|---|---|---|---|---|---|
| | +ve | −ve | +ve | −ve | N | Ab |
| Age (years) | | | | | | |
| <40 | 1 | 0 | 1 | 0 | 1 | 0 |
| 40–59 | 23 | 12 | 35 | 0 | 18 | 17 |
| 60–79 | 97 | 68 | 151 | 14 | 54 | 111 |
| 80–100 | 31 | 18 | 44 | 5 | 14 | 35 |
| | $\chi^2$=1.40 $P$=0.704 | | $\chi^2$=3.62 $P$=0.306 | | $\chi^2$=7.29 $P$=0.063 | |
| Sex | | | | | | |
| Male | 74 | 49 | 110 | 13 | 40 | 83 |
| Female | 78 | 49 | 121 | 6 | 47 | 80 |
| | $\chi^2$=0.041 $P$=0.839 | | $\chi^2$=0.081 $P$=0.0813 | | $\chi^2$=0.554 $P$=0.456 | |
| Site | | | | | | |
| Caecum | 21 | 13 | 33 | 1 | 10 | 24 |
| Ascending colon | 20 | 14 | 29 | 5 | 11 | 23 |
| Transverse colon | 18 | 15 | 32 | 1 | 13 | 20 |
| Descending colon | 4 | 3 | 7 | 0 | 3 | 4 |
| Sigmoid colon | 40 | 21 | 56 | 5 | 26 | 35 |
| Rectum | 49 | 32 | 74 | 7 | 24 | 57 |
| | $\chi^2$=1.24 $P$=0.941 | | $\chi^2$=5.21 $P$=0.39 | | $\chi^2$=3.63 $P$=0.604 | |
| Tumour size (cm) | | | | | | |
| <2.5 | 65 | 37 | 94 | 8 | 37 | 65 |
| 2.5–4.9 | 67 | 42 | 101 | 8 | 36 | 73 |
| 5.0–7.4 | 14 | 10 | 23 | 1 | 8 | 16 |
| 7.5–9.9 | 5 | 8 | 12 | 1 | 5 | 8 |
| 10.0–12.4 | 1 | 0 | 1 | 0 | 1 | 0 |
| 12.5–15.0 | 0 | 1 | 0 | 1 | 0 | 1 |
| | $\chi^2$=5.36 $P$=0.373 | | $\chi^2$=12.7 $P$=0.027 | | $\chi^2$=2.75 $P$=0.738 | |
| Tumour type | | | | | | |
| Adenocarcinoma NOS | 141 | 82 | 206 | 17 | 80 | 143 |
| Mucinous | 9 | 16 | 23 | 2 | 6 | 19 |
| Other | 2 | 0 | 2 | 0 | 1 | 1 |
| | $\chi^2$=8.29 $P$=0.016 | | $\chi^2$=0.170 $P$=0.918 | | $\chi^2$=1.60 $P$=0.448 | |
| Tumour grade | | | | | | |
| Well differentiated | 24 | 16 | 37 | 3 | 16 | 24 |
| Mod differentiated | 89 | 52 | 129 | 12 | 46 | 95 |
| Poorly differentiated | 39 | 30 | 65 | 4 | 25 | 44 |
| | $\chi^2$=0.860 $P$=0.651 | | $\chi^2$=0.486 $P$=0.784 | | $\chi^2$=0.833 $P$=0.659 | |
| Lymphocytes | | | | | | |
| Prominent | 22 | 9 | 14 | 5 | 10 | 21 |
| Not prominent | 130 | 89 | 205 | 26 | 77 | 142 |
| | $\chi^2$=1.53 $P$=0.215 | | $\chi^2$=3.67 $P$=0.056 | | $\chi^2$=0.101 $P$=0.751 | |
| Tumour margin | | | | | | |
| Pushing | 78 | 55 | 120 | 13 | 48 | 85 |
| Infiltrating | 74 | 43 | 111 | 6 | 39 | 78 |
| | $\chi^2$=0.553 $P$=0.457 | | $\chi^2$=1.91 $P$=0.166 | | $\chi^2$=0.208 $P$=0.648 | |
| Vascular invasion | | | | | | |
| None | 87 | 56 | 130 | 13 | 47 | 96 |
| Present | 65 | 42 | 101 | 6 | 40 | 67 |
| | $\chi^2$=0.00021 $P$=0.988 | | $\chi^2$=1.057 $P$=0.304 | | $\chi^2$=0.550 $P$=0.458 | |
| Dukes' stage | | | | | | |
| A | 18 | 11 | 25 | 4 | 10 | 19 |
| B | 49 | 39 | 81 | 7 | 31 | 57 |
| C1 | 73 | 42 | 108 | 7 | 44 | 71 |
| C2 | 12 | 6 | 17 | 1 | 2 | 16 |
| | $\chi^2$=1.59 $P$=0.661 | | $\chi^2$=2.08 $P$=0.556 | | $\chi^2$=5.06 $P$=0.167 | |

Rb1 +ve or −ve indicates overall positive or negative Rb1 staining. N, normal staining; Ab, aberrant staining.

differentiation), degree of intratumoral fibrosis, presence or absence of a prominent tumour lymphocytic infiltrate, nature of the invasive tumour margin (pushing or infiltrating) and presence of extramural vascular or lymphatic invasion. The number of lymph nodes containing metastatic carcinoma, high lymph node involvement adjacent to the apical vascular tie (indicating Dukes' C2 stage), local peritoneal infiltration by tumour, Dukes' stage (A, B, C1 or C2), Jass grade and Australian Clinicopathological Staging systems were also assessed (Shepherd et al, 1995). For the purposes of the current study, representative archival tumour blocks were selected for 250 consecutive patients early in the study to maximize the length of follow-up.

### Immunohistochemistry

Representative blocks of formalin-fixed paraffin-embedded tumour tissue were cut at 4-μm thickness, dewaxed in xylene and then rinsed in alcohol and graded alcohol/water mixtures. A standard avidin/biotin peroxidase technique was used. Incubation with the primary p53 antibody D0-1 (Vojtesek et al, 1992) (Professor DP Lane, Cancer Research Campaign Molecular Oncogenesis Unit, University Of Dundee) was performed for 30 min at a dilution of 1:100. D0-1 is a well-characterized monoclonal antibody that recognizes an epitope near the N-terminus of both wild and mutant p53 proteins (Vojtesek et al, 1992). Following incubation with the primary antibody, a secondary polyclonal biotinylated swine anti-rabbit antibody was used (Dako UK, High Wycombe, Bucks, UK). This was followed by an avidin–biotinylated peroxidase complex, with 3,3-diaminobenzidene as the chromogen. For the detection of Rb1 protein, the monoclonal antibody 14,0001A was used (PharMingen, San Diego, CA, USA) at a dilution of 1:100, followed by a secondary polyclonal biotinylated swine anti-rabbit antibody (Dako UK) with an avidin–biotinylated peroxidase complex, with 3,3-diaminobenzidene as the chromogen.

### Scoring and interpretation of results

All sections were incubated in batches of 30–40 tumours with a tumour of known positive immunoreactivity with each run of immunostaining. Only nuclear staining of tumour nuclei was interpreted to indicate positive tumour p53 or Rb1 immunoreactivity. All tumour sections were examined by DNP. The percentage of tumour nuclei staining across the whole tumour section was recorded subjectively in five categories as follows: 0%, 1–24%, 25–49%, 50–74%, 75–100%; and the grading of the intensity of immunostaining was recorded in four categories (0, 1, 2, 3), with 0 indicating no nuclear staining, 1 very weak nuclear staining, 2 intermediate nuclear staining and 3 strong nuclear staining. The score for intensity was always that of the most strongly stained

**Table 2** Local recurrence p53 and Rb1 staining

| | p53 | | Rb1 | |
|---|---|---|---|---|
| | +ve | −ve | +ve | −ve |
| No evidence of recurrence | 126 | 81 | 192 | 15 |
| Local recurrence | 26 | 17 | 39 | 4 |
| | $\chi^2$=0.0024 $P$=0.960 | | $\chi^2$=0.643 $P$=0.214 | |

© Cancer Research Campaign 1997

nucleus in any section, not the median intensity of nuclear staining. In occasional cases of doubt as to the interpretation of staining results, a joint decision was made with NAS after examination of tumour sections using a conference microscope. For the purposes of statistical analysis, p53 expression was treated as positive if any tumour showed nuclear staining for p53; and for Rb1, a positive result was noted if any tumour nucleus showed expression of Rb1 protein. Aberrant Rb1 expression, for the purposes of this study, was defined as any tumour showing less than 50% of tumour nuclei with Rb1 protein expression.

### Statistical analysis

Data were analysed using Statistica, a computer software package for a Windows IBM-compatible personal computer. The recurrence-free survival and overall survival analyses were performed using the log-rank test (Mantel, 1966). Multivariate analyses were performed using the Cox proportional hazards model (Cox, 1972).

### RESULTS

### p53 immunostaining

The results of p53 immunostaining and clinical variables are shown in Tables 1 and 2 and Figure 1. There was little or no evidence of cytoplasmic staining for p53 with D0-1. No staining of normal crypt epithelial cells was identified, although very weak staining of basal crypt epithelial cells was visible in some cases in transitional mucosa adjacent to tumours. One hundred and fifty-two of 250 (60.8%) cases showed positive tumour p53 staining (Table 1). The pattern of staining was variable between tumours, some showing strong generalized nuclear immunoreactivity for p53 throughout the tumour sections, whereas others did not. Some showed larger nuclei with strong focal p53 staining. There was no relationship between tumour p53 expression and patient age, sex, site of tumour, tumour size, tumour type (standard adenocarcinoma or mucinous), tumour grade, peritumoral lymphocytic infiltrate, nature of the invasive tumour margin, extramural vascular or lymphatic invasion, lymph node involvement, high tie lymph node involvement, involvement of the peritoneum by tumour and Dukes' stage. There was no relationship of tumour p53 expression with recurrence-free survival or overall patient survival (Figure 2). There was a direct relationship between p53 and Rb1 staining (Table 2).

### Rb 1 immunostaining

Positive nuclear Rb1 staining was seen in the majority of tumours examined – 207 of 250 (82.8%) cases (Table 1 and Figure 3). No cytoplasmic staining was seen for Rb1. Positive Rb1 staining was also seen in the nuclei of crypt cells of transitional mucosa adjacent to the invasive tumours. There was again no relationship between tumour Rb1 protein expression and patient age, sex, site of tumour, tumour size, tumour type (standard adenocarcinoma or mucinous), tumour grade, peritumoral lymphocytic infiltrate, nature of the invasive tumour margin, extramural vascular or lymphatic invasion, lymph node involvement, involvement of the peritoneum by tumour and Dukes' stage. There was no relationship between tumour Rb1 expression and recurrence-free survival or overall patient survival (Figures 4 and 5). Reduced or 'aberrant' Rb1 staining was also examined. This showed that the group of tumours with less than 50%

**Figure 1** Tumour nuclei staining strongly (intensity score=3) with antibody DO-1 (× 190 magnification)

**Figure 2** p53 status and overall survival. $Z=-0.926$, $P=0.177$ (NS)

Rb1 staining appeared in univariate analysis to have reduced overall and recurrence-free survival. However, in multivariate Cox proportional hazards analysis, the prognostic effect of aberrant Rb1 protein expression failed to achieve independent prognostic significance.

© Cancer Research Campaign 1997

**Table 3** p53 and Rb1 expression

| Rb1 | p53 | |
|---|---|---|
| | +ve | −ve |
| Rb1 −ve | 6 | 13 |
| Rb1+ve | 146 | 85 |
| | $\chi^2$=7.37 $P$=<0.01 | |

**Figure 3** Tumour nuclei staining focally and strongly (intensity score=3) with antibody 14,001A (x235 magnification)

**Figure 4** Tumour Rb1 status and overall survival (any tumour nucleus +ve compared with tumours showing all nuclei with −ve staining). $Z$=0.0148, $P$=0.494 (NS)

**Figure 5** Tumour Rb1 status and aberrant Rb1 expression. $Z$=− 1.82, $P$=0.034 (Significant)

### DISCUSSION

This is one of the largest studies to assess the prognostic implication of p53 expression in colorectal cancer. The results show that there was little or no prognostic effect of p53 and Rb1 protein expression in a large unselected series of colorectal carcinomas. This finding accords with some recent reports in the literature but not with other previously published series. A novel relationship between p53 and Rb1 protein expression has been identified in this study. It was initially suggested that detection of p53 protein correlated with *p53* gene mutations (Iggo et al, 1990). However, there are a large number of pathological studies in which immunohistochemical p53 protein analysis has been examined together with *p53* gene mutations in a variety of tumour types (Dunn et al, 1993; Baas et al, 1994, 1996; McManus et al, 1994; Kocialkowski et al, 1995; Nylander et al, 1995). It has been stated that mutation of the *p53* gene is one of the commonest mutations in human cancers, although mutations of *p53* occur late in the adenoma–carcinoma sequence of colorectal cancer (Fearon and Vogelstein, 1990). *p53* mutations may, however, occur early in some pathways and later in other tumour types. Expression of p53 may occur in a considerable number of cases in which *p53* gene mutations are not detectable by polymerase chain reaction (PCR) (Baas et al, 1994, 1996). This again is not surprising; p53 is a normal constitutive cell cycle-associated protein, and the ability to detect p53 protein in archival formalin-fixed paraffin-embedded tissue is dependent on the sensitivity of the antigen retrieval system employed. The microwave antigen retrieval system used for detection of p53 and Rb1 in the present study is quite likely to have improved the sensitivity of p53 and Rb1 antigen detection (McKee et al, 1993,

© Cancer Research Campaign 1997

**Table 4** p53 and prognosis in colorectal carcinoma

| Reference | Number of cases in series | p53 poor prognostic factor | Multivariate analysis Yes/No and other comments |
|---|---|---|---|
| Scott et al (1991) | 52 | N | No MVA. Small series, no effect of p53 on survival |
| Remvikos et al (1992) | 78 | Y | No MVA. No effect of p53 on survival when Dukes' 'D' cases excluded |
| Starzynska et al (1992) | 107 | Y | No MVA. One year clinical follow-up, adverse survival in group of p53 +ve cases |
| Sun et al (1992) | 293 | Y | MVA – yes. Cytoplasmic *not* nuclear p53 expression associated with adverse survival |
| Yamaguchi et al (1992) | 100 | Y | MVA – yes. Three-year survival significantly worse for p53-expressing cases |
| Bell et al (1993) | 100 | N | No MVA. No effect of p53 on survival except in ten cases also showing Ki *ras* mutations |
| Sun et al (1993) | 293 | Y | MVA – yes. Nuclear and cytoplasmic p53 expression associated with adverse prognosis in Dukes' A, B and C tumours |
| Yamaguchi et al (1993) | 203 | Y | No MVA. p53-positive cases associated with adverse 5-year survival |
| Auvinen et al (1994) | 144 | Y | No MVA. Overall survival reduced in p53+ve tumours |
| Goh et al (1994) | 187 | Y | No MVA. PCR and immuno-histochemical study showing that lymphatic dissemination associated with *p53* mutation |
| Hamelin et al (1994) | 85 | Y | MVA – yes. PCR study showing adverse effect of p53 mutations on survival (median 47 months) |
| Nathanson et al (1994) | 84 | N | MVA – yes. No statistically significant relationship of p53 and overall survival identified |
| Zeng et al (1994) | 107 | Y | MVA – yes. Study conducted on Dukes' C or above patients with low CEA levels |
| Bertorelle et al (1995) | 83 | Y | No MVA. Increased frequency of liver and nodal metastases in p53+ve tumours. Overall survival was not assessed |
| Mulder et al (1995) | 109 | N | MVA – yes. p53 expression not an independent marker of prognosis |
| Ofner et al (1995) | 109 | N | No MVA. p53 expression not an independent marker of prognosis |

Baas et al, 1996), implying that a considerable proportion of p53-staining cases would show absence of p53 mutations. Equally, cases 'aberrantly staining' for Rb1 might also not harbour *Rb1* gene mutations.

Other studies have examined the prognostic effect of p53 protein expression in colorectal cancer (see Table 4), and most would suggest that expression of p53 is an adverse prognostic factor. The immunohistochemical evidence for a relationship between p53 expression and adverse prognosis has been corroborated either by studies of total tumour p53 or by PCR analysis of *p53* gene mutations (see Table 4).

One conflicting study showed that a group of patients with either nil or strong p53 protein expression fared worse than patients showing intermediate staining for p53 (Nathanson et al, 1994). It has also been suggested that p53 protein expression in the cytoplasm rather than the nucleus itself may be a predictor of poor prognosis in colorectal cancer, and that nuclear p53 staining is not an adverse prognostic factor (Sun et al, 1992). Thirty of 293 cases of colorectal carcinoma showed pure cytoplasmic p53 expression. This particular study was, however, performed with a polyclonal antibody, CM 1, and the number of cases showing pure cytoplasmic staining was small. The same authors also showed that both nuclear and p53 protein expression appeared to be important in a multivariate analysis (Sun et al, 1993). A further study (Ofner et al, 1995) showed that there was no relationship between p53 protein expression and grade or stage parameters, such as Dukes' stage, in colorectal cancer. This finding has been reiterated (Mulder et al, 1995) in a study of 109 colorectal cancers. p53 expression was found to be more frequent in non-mucinous tumours and in metastatic tumours than in primary carcinomas.

*© Cancer Research Campaign 1997*

Dukes' stage and pathological grade were independent prognostic variables, whereas p53 was not an independent prognostic marker.

To our knowledge, this is one of the largest studies to assess the prognostic implications of p53 expression in colorectal cancer. These results show that there is no prognostic effect of p53 and Rb1 protein expression. This finding accords with some reported results in the literature but not others. A relationship between p53 and Rb1 protein expression was identified; this finding is novel. There is little if any published literature on Rb1 and prognosis in colorectal cancer. The expression of Rb1 protein was examined in 50 cases of colorectal cancer showing localization of the Rb1 protein to the nucleus of tumour cells (Yamaoto et al, 1995). Strong expression was seen in 17 cases, intermediate expression in 22 cases and weak (up to 10% of the nuclei staining) in 11 cases. The lack of association of Rb1 staining and other prognostic factors in colorectal cancer is not particularly surprising. The Rb1 protein is thought to play a role in the $G_1$ phase and mutations of the Rb1 protein are thought to be important in tumorigenesis of second tumours, in particular sarcomas, in patients with retinoblastoma gene mutations. The idea that lack of expression of the Rb1 protein (which should, by inference, be associated with *Rb1* gene 'double knockout') would be an adverse prognostic factor in colorectal cancer has not been examined. It is interesting to contrast our findings in colorectal cancers with those in sarcomas. Adverse prognosis has been associated with reduced or 'aberrant' expression of Rb1 protein in tumours with heterogeneous or absent expression of the *Rb1* gene product (Cance et al, 1990).

The results from this study imply that expression of p53 and Rb1 proteins does not appear to be a useful prognostic factor in colorectal cancer while coexpression of p53 and Rb1 proteins is present in colorectal cancer. The apparent discrepancy of published results in the literature may to some extent reflect publication biases particularly as many studies show an effect of p53 on overall prognosis but not on other important prognostic parameters, such as tumour stage, or in subsets of particular patients in any given study but not in whole cohorts of patients.

### ACKNOWLEDGEMENTS

The authors are grateful to South and West Regional Health Authority, which partially funded this research project (project grant no. 296), to the Imperial Cancer Research Fund and Professor NA Wright for financial support for technical assistance (KJB), to Professor DP Lane at the Cancer Research Campaign Molecular Oncogenesis Unit, University of Dundee, for provision of D0-1 antibody, and for the co-operation and diligence of surgical colleagues at Gloucestershire Royal Hospital and general practioners in West Gloucestershire. We are also indebted to MLSO staff, particularly C Hackwood, J Rachel, and M Ralphs.

### REFERENCES

Auvinen A, Isola J, Visakorpi T, Koivula T, Virtanen S and Hakama M (1994) Overexpression of p53 and long-term survival in colon carcinoma. *Br J Cancer* **70**: 293–296

Baas IO, Mulder JW, Offerhaus GJ, Vogelstein B and Hamilton SR (1994) An evaluation of six antibodies for immunohistochemistry of mutant p53 gene product in archival colorectal neoplasms. *J Pathol* **172**: 5–12

Baas IO, Van Den Berg FM, Mulder JW, Clement MJ, Slebos RJC, Hamilton SR and Offerhaus GJA (1996) Potential false-positive results with antigen enhancement for immunohistochemistry of the p53 gene product in colorectal neoplasms. *J Pathol* **178**: 264–267

Bell SM, Scott N, Cross D, Sagar P, Lewis FA, Blair GA, Taylor GR, Dixon MF and Quirke P (1993) Prognostic value of p53 overexpression and c-Ki-ras gene mutations in colorectal cancer. *Gastroenterology* **104**: 57–64

Bertorelle R, Esposito G, Mistro AD, Belluco C, Nitti D, Lise M and Bianchi LC (1995) Association of p53 gene and protein alterations with metastases in colorectal cancer. *Am J Surg Pathol* **19**: 463–471

Cance WG, Brennan MF, Dudas ME, Huang CM and Cardo CC (1990) Altered expression of the retinoblastoma gene product in human sarcomas. *N Engl J Med* **323**: 1457–1462

Cox DR (1972) Regression models and life tables. *JR Stat Soc B* **34**: 187–220

Dunn JM, Hastrich DJ, Newcomb P, Webb JC, Maitland NJ and Farndon JR (1993) Correlation between p53 mutations and antibody staining in breast carcinoma. *Br J Surg* **80**: 1410–1413

Fearon ER and Vogelstein B (1990) A genetic model for colorectal tumourigenesis. *Cell* **61**: 759–767

Goh HS, Chan CS, Khine K and Smith DR (1994) p53 and behaviour of colorectal cancer. *Lancet* **344**: 233–234

Hamelin R, Puig-Laurent P, Olschwang S, Jego N, Asselain B, Remvikos Y, Girodet J, Salmon RJ and Thomas G (1994) Association of p53 mutations with short survival in colorectal cancer. *Gastroenterology* **106**: 42–48

Iggo R, Gatter K, Bartek J, Lane D and Harris AL (1990) Increased expression of mutant forms of p53 oncogene in primary lung cancer. *Lancet* **335**: 675–679

Kern SE, Pietenpol JA, Thiagalingam S, Seymour A, Kinzler KW and Vogelstein B (1992) Oncogenic forms of p53 inhibit p53-regulated gene expression. *Science* **256**: 827–830

Kocialkowski S, Pezzella F, Morrison H, Jones M, Laha S, Harris AL, Mason DY and Gatter KC (1995) Mutations in the p53 gene are not limited to classic 'hot spots' and are not predictive of p53 protein expression in high-grade non-Hodgkin's lymphoma. *Br J Haematol* **89**: 55–60

Lemoine NR (1994) Molecular biology of breast cancer. *Ann Oncol* **4**: 31–37

Mantel N (1996) Evaluation of survival time data and two new rank order statistics arising in its consideration. *Cancer Chemother Rep* **50**: 163–170

McKee PH, Hobbs C and Hall PA (1993) Antigen retrieval by microwave irradiation lowers immunohistological detection thresholds. *Histopathology* **23**: 377–379

McManus DT, Yap EP, Maxwell P, Russell SE, Toner PG and McGee JO (1994) p53 expression, mutation, and allelic deletion in ovarian cancer. *J Pathol* **174**: 159–168

Michalovitz D, Halevy O and Oren M (1990) Conditional inhibition of transformation and of cell proliferation by a temperature sensitive mutant of p53. *Cell* **62**: 671–680

Mulder JWR, Baas IO, Polak MM, Goodman SN and Offerhaus GJA (1995) Evaluation of p53 protein expression as a marker for long term prognosis in colorectal carcinoma. *Br J Cancer* **71**: 1257–1262

Nathanson SD, Linden MD, Tender P, Zarbo RJ, Jacobsen G and Nelson LT (1994) Relationship among p53, stage, and prognosis of large bowel cancer. *Dis Colon Rectum* **37**: 527–534

Nylander K, Nilsson P, Mehle C and Roos G (1995) p53 mutations, protein expression and cell proliferation in squamous cell carcinomas of the head and neck. *Br J Cancer* **71**: 826–830

Ofner D, Maier H, Riedmann B, Holzberger P, Nogler M, Totsch M, Bankfalvi A, Winde G, Bocker W and Schmid KW (1995) Immunohistochemically detectable p53 and mdm-2 oncoprotein expression in colorectal carcinoma: prognostic significance. *J Clin Pathol, Mol Pathol* **48**: 12–16

Remvikos Y, Tominaga O, Hammel P, Puig-Laurent P, Salmon RJ, Dutrillaux B and Thomas G (1992) Increased p53 protein content of colorectal tumours correlates with poor survival. *Br J Cancer* **66**: 758–764

Scott N, Sagar P, Stewart J, Blair GE, Dixon MF and Quirke P (1991) p53 in colorectal cancer: clinicopathological correlation and prognostic significance. *Br J Cancer* **63**: 317–319

Shepherd NA, Baxter KJ and Love SB (1995) Influence of local peritoneal involvement on pelvic recurrence and prognosis in rectal cancer. *J Clin Pathol* **48:** 849–855

Sherr CJ (1994) Growth factor-regulated G1 cyclins. *Stem Cells* **12**: 47–55

Smith ML, Chen IT, Zhan Q, Bae I, Chen CY, Gilmer TM, Kastan MB, O'Connor PM and Fornace AJ (1994) Interaction of the p53-regulated protein Gadd45 with proliferating cell nuclear antigen. *Science* **266**: 1376–1380

Starzynska T, Bromley M, Ghosh A and Stern PL (1992) Prognostic significance of p53 overexpression in gastric and colorectal cancer. *Br J Cancer* **66**: 558–562

Sun XF, Carstensen JM, Zhang H, Stal O, Wingren S, Hatschek T and Nordenskjold B (1992) Prognostic significance of cytoplasmic p53 oncoprotein in colorectal adenocarcinoma. *Lancet* **340**: 1369–1373

Sun XF, Carstensen JM, Stal O, Zhang H, Nilsson E, Sjodahl R and Nordenskjold B (1993) Prognostic significance of p53 expression in relation to DNA ploidy in colorectal adenocarcinoma. *Virchows Archiv A Pathol Anat* **423**: 443–448

© Cancer Research Campaign 1997

Vojtesek B, Bartek J and Midgeley CA (1992) An immunochemical analysis of the human nuclear phosphoprotein p53: new monoclonal antibodies and epitope mapping using recombination p53. *J Immunol Methods* **151**: 237–242

Yamaguchi A, Kurosaka Y, Fushida S, Kanno M, Yonemura Y, Miwa K and Miyazaki I (1992) Expression of p53 protein in colorectal cancer and its relationship to short term prognosis. *Cancer* **70**: 2778–2784

Yamaguchi A, Nakagawara G, Kurosaka Y, Nishimura G, Yonemura Y and Miyazaki I (1993) p53 immunoreaction in endoscopic biopsy specimens of colorectal cancer and its prognostic significance. *Br J Cancer* **68**: 399–402

Yamamoto H, Monden T, Ikeda K, Izawa H, Fukuda K, Fukunaga M, Tomita N, Shimano T, Shiozaki H and Monden M (1995) Co-expression of cdk/cdc2 and retinoblastoma gene products in colorectal cancer. *Br J Cancer* **71**: 1231–1236

Zeng ZS, Sarkis AS, Zhang ZF, Klimstra DS, Charytonowicz E, Guillem JG, Cardo CC and Cohen AM (1994) p53 nuclear overexpression: an independent predictor of survival in lymph node-positive colorectal cancer patients. *J Clin Oncol* **12**: 2043–2050

© Cancer Research Campaign 1997